# The application value of LAVA-flex sequences in enhanced MRI scans of nasopharyngeal carcinoma: comparison with T1WI-IDEAL

**DOI:** 10.3389/fonc.2024.1320280

**Published:** 2024-02-14

**Authors:** Li Peng, Bijuan Chen, Erhan Yu, Yifei Lin, Jiahao Lin, Dechun Zheng, Yu Fu, Zhipeng Chen, Hanchen Zheng, Zhouwei Zhan, Yunbin Chen

**Affiliations:** ^1^ Department of Radiology, Clinical Oncology School of Fujian Medical University, Fujian Cancer Hospital, Fuzhou, Fujian, China; ^2^ Department of Radiation Oncology, Clinical Oncology School of Fujian Medical University, Fujian Cancer Hospital, Fuzhou, Fujian, China; ^3^ Department of Neurology, Fujian Medical University Union Hospital, Fuzhou, Fujian, China; ^4^ School of Basic Medical Sciences of Fujian Medical University, Fuzhou, Fujian, China; ^5^ Department of Medical Oncology, Clinical Oncology School of Fujian Medical University, Fujian Cancer Hospital, Fuzhou, Fujian, China

**Keywords:** nasopharyngeal carcinoma, MRI imaging, LAVA-Flex, T1WI-IDEAL, fat suppression

## Abstract

**Introduction:**

Magnetic resonance imaging (MRI) staging scans are critical for the diagnosis and treatment of patients with nasopharyngeal cancer (NPC). We aimed to evaluate the application value of LAVA-Flex and T1WI-IDEAL sequences in MRI staging scans.

**Methods:**

Eighty-four newly diagnosed NPC patients underwent both LAVA-Flex and T1WI-IDEAL sequences during MRI examinations. Two radiologists independently scored the acquisitions of image quality, fat suppression quality, artifacts, vascular and nerve display. The obtained scores were compared using the Wilcoxon signed rank test. According to the signal intensity (SI) measurements, the uniformity of fat suppression, contrast between tumor lesions and subcutaneous fat tissue, and signal-to-noise ratio (SNR) were compared by the paired t-test.

**Results:**

Compared to the T1WI-IDEAL sequence, LAVA-Flex exhibited fewer artifacts (*P*<0.05), better visualization of nerves and vessels (*P*<0.05), and performed superior in the fat contrast ratio of the primary lesion and metastatic lymph nodes (0.80 *vs*. 0.52, 0.81 *vs*. 0.56, separately, *P*<0.001). There was no statistically significant difference in overall image quality, tumor signal-to-noise ratio (SNR), muscle SNR, and the detection rate of lesions between the two sequences (*P*>0.05). T1WI-IDEAL was superior to LAVA-Flex in the evaluation of fat suppression uniformity (*P*<0.05).

**Discussion:**

LAVA-Flex sequence provides satisfactory image quality and better visualization of nerves and vessels for NPC with shorter scanning times.

## Introduction

Nasopharyngeal carcinoma (NPC) exhibits a distinctive geographical distribution, primarily affecting East and Southeast Asia. Within China, NPC stands as one of the most prevalent malignant tumors in the head and neck region. Incidence and mortality rates in China surpass the global average, particularly in southern provinces like Guangdong, Guangxi, Jiangxi, Hunan, and Fujian (1, 2). Early symptoms of NPC include nasal congestion, blood in nasal mucus, ear stuffiness, and headaches. These manifestations often resemble those of rhinitis, making them easily overlooked. However, delayed diagnosis and treatment can lead to further complications such as hearing disorders (e.g., tinnitus or unilateral/bilateral hearing loss) or tumor metastasis. Ultimately, these complications can result in lesions in other organs and systems within the body, posing a significant threat to patients’ lives (3, 4). In regions with a high prevalence of NPC or for individuals with a family history of the disease, it is crucial to undergo regular screening tests for NPC. These may include nasal endoscopy, CT or MR scans of the nasopharynx, and EB virus-associated antibody tests, among others. Such screenings play a vital role in achieving early detection of NPC (5).

Since the release of the 2008 edition of Chinese NPC staging, magnetic resonance imaging (MRI) has been recommended as the primary imaging technique for T and N staging of NPC (6). The nasopharyngeal structures are intricate and delicate, with a wide range of potential lesion involvement. MRI offers high soft tissue resolution, multi-parameter imaging, and arbitrary orientation capabilities, allowing for a more accurate assessment of primary tumor invasion depth, lymph node metastasis areas, and more. Thus, MRI plays a crucial role in diagnosing nasopharyngeal tumors and improving the early detection rate of NPC. In the diagnosis and T/N staging of NPC, MRI enhancement scanning combined with fat suppression is an essential component sequence. The conventional fat suppression technique used in MRI scanning protocols for NPC, like 2D FSE TIWI FS sequence, has some limitations, such as uneven or failed fat suppression, which cannot meet the diagnostic needs (7, 8). Another sequence, DCE-MRI, is based on conventional MRI and employs fast MRI acquisition technology to complete multi-phase magnetic resonance scanning in a short period. However, there are challenges associated with this sequence, including poor image signal-to-noise ratio and unclear lesion boundaries, which prevent its routine use (9). To overcome this, various improved fat suppression imaging techniques based on the Dixon water and fat separation technique have been developed. Examples include the Iterative Decomposition of Water and Fat using Echo Asymmetry and Least Squares Estimation (IDEAL) and the Flex technique.

In this study, we conducted a comparative analysis of two enhanced sequences: 2D T1WI-IDEAL (10) and 3D Liver Volume Accelerated Flex Acquisition (LAVA Flex) (11). LAVA Flex technology seamlessly integrates a cutting-edge phase correction with a symmetric two-point signal acquisition approach. It leverages two distinct TEs (echo times) to capture signals from both water and fat, enabling the computation of in-phase and out-of-phase images. This innovative technique can be achieved within a significantly reduced TR (repetition time), enhancing both the speed and efficiency of imaging (12–14). Notable differences exist between these two sequences in terms of imaging technique, slice thickness, fat suppression method, and scanning duration. Specifically, the T1WI-IDEAL sequence operates as a rapid spin echo sequence, whereas the LAVA-FLEX sequence functions as a rapid gradient echo sequence, falling under the category of volume scanning. Furthermore, the T1WI-IDEAL sequence exhibits a slice thickness of 5mm with a 1mm interval, while the LAVA-FLEX sequence boasts a slice thickness of 1mm without any interval. Both sequences leverage modified Dixon techniques for fat suppression, albeit with distinct implementations. Notably, the T1WI-IDEAL sequence necessitates scanning all axes to complete a comprehensive staging scan for NPC, resulting in an approximate duration of 6 minutes. Conversely, the LAVA-FLEX sequence requires scanning in only one direction, with the remaining two directional images being generated through post-processing, thereby optimizing time efficiency (11). This is particularly advantageous for elderly patients and those exhibiting severe clinical symptoms who may have limited tolerance for prolonged examinations. The application of the LAVA-FLEX sequence in such contexts holds significant clinical value. Previous studies have demonstrated the superiority of LAVA-Flex sequence imaging over other conventional scanning sequences, including the T1WI-IDEAL sequence (8, 15–17). However, no studies have yet reported a comparative analysis of imaging quality between LAVA-Flex and T1WI-IDEAL sequences in NPC.

Therefore, this study aims to assess the effectiveness of LAVA-flex and compare the quality of water-only images generated by LAVA-flex with those obtained from the T1WI-IDEAL sequence in patients with NPC.

## Materials and methods

### Patients

This study was conducted following the approval of Fujian Cancer Hospital (No: K2021-048-01). All enrolled patients provided informed consent by signing an informed consent form. The study included newly diagnosed NPC patients who were scheduled for initial treatment at our hospital between July 2020 and April 2023. MRI scans of the nasopharynx and neck were performed on these patients, utilizing both LAVA-Flex and T1WI-IDEAL enhanced scans. The inclusion criteria for this study were as follows: (1) Age over 18 years; (2) Patients with newly diagnosed NPC confirmed by pathology; (3) Patients who agreed to undergo MRI-enhanced examination and provided informed consent. Patients who had previously received treatments such as chemotherapy, radiotherapy, targeted therapy, or other therapies were excluded from the study. Additionally, patients with contraindications to MR imaging (such as metal implants, magnetic implants, claustrophobia, removable dentures, etc.), a history of allergy to contrast agents used in MR imaging, or severe bronchial asthma were also excluded. A total of 84 patients met the inclusion and exclusion criteria and were selected for this study. Among them, there were 66 males and 18 females, with an average age of 48.6 ± 11.9 years (ranging from 19 to 79 years). Biopsy results confirmed that all nasopharyngeal lesions in the selected patients were classified as either non-keratinizing undifferentiated carcinoma in 82 cases or non-keratinizing differentiated carcinoma in two cases.

### MRI technique

In this study, all cases underwent MRI examinations using a 3.0T superconducting magnetic resonance imaging system (Discovery MR750W 3.0T, GE Healthcare) equipped with a 12-channel head and neck coil. Before the examination, any removable dentures and metal objects on the body were carefully removed to ensure accurate imaging results. During the examination, patients were positioned in a supine position with their heads advanced and their shoulders pressed against the head and neck coil. Cushions were placed on both sides of the patient’s head to immobilize it and minimize movement during the scan. The nasal bridge was adjusted to be vertical concerning the bed surface for proper alignment. The center of the scan was positioned at the tip of the nose, and scans were performed while patients maintained a relaxed and steady breathing state. This approach helped reduce motion artifacts and improve image quality. The MRI sequences used in this study included T1WI-IDEAL and LAVA-Flex enhanced scans, as well as other conventional sequences commonly employed for NPC imaging. These sequences provided comprehensive information for accurate diagnosis and staging purposes. For detailed scanning parameters, please refer to **Table 1** below.

**Table 1:** The detailed parameters of each scanning sequence.

	LAVA-Flex+C	Ax T1WI-IDEAL+C	Cor T1WI-IDEAL+C	Sag T1WI-IDEAL+C
TR (ms)	8.5	754	715	536
TE (ms)	1.3	9.4	Min Full	Min Full
Section thickness (mm)	1	5	5	5
Intersection gap (mm)		1	1	1
BW	142.86	62.5	83.33	83.33
FOV (mm×mm)	300×300	240×240	300×300	240×240
Matrix	300×300	288×224	288×224	288×224
NEX	1	1	1	1
Acquisition time(s)	245	214	95	65

TR, repetition time; TE, echo time; BW, bandwidth; FOV, field of view; NEX, number of excitations.

### MRI analysis

All images were transmitted and stored in DICOM format to the PACS (Picture Archiving and Communication Systems) and then transferred to the post-processing workstation (AW Volume Share 7, GE MEDICAL SYSTEMS SCS) for data processing. Two radiologists (YC and DZ), each with more than 15 years of experience in head and neck radiology, independently evaluated all images. The radiologists were not able to be blinded to the type of acquisition due to the obvious appearance differences between the images generated by different sequences. However, they were unaware of the endpoint of the study. After the MRI scans, all images were converted and stored in DICOM format, which is a standard for medical imaging data. These DICOM images were then transmitted to the PACS for storage and management. Subsequently, the images were transferred to a dedicated post-processing workstation (AW Volume Share 7, GE MEDICAL SYSTEMS SCS) for further analysis and processing of data. To ensure a reliable and accurate evaluation of the images, two experienced radiologists (YC and DZ) with over 15 years of expertise in head and neck radiology independently reviewed them. Although blinding them to the type of acquisition was not feasible due to distinct appearance differences between sequences, it’s important to note that both radiologists remained unaware of the study’s endpoint during their evaluation process.

### Qualitative image analysis

The overall image quality (18), uniformity of fat suppression (12), artifacts (8), tumor conspicuity (19), and vascular display (20) for both sequences were comparatively assessed by two radiologists (YC and DZ) using respective scales. All image quality scores were determined through consultation between the two radiologists. Additionally, the presentation of different sequences at the tumor invasion site was further compared and analyzed, including cervical lymph nodes, retropharyngeal lymph nodes, cervical spine, skull base bone, sphenoid sinus, ethmoid sinus, parapharyngeal space, medial and lateral pterygoid muscles, pterygomaxillary fissure, nasal cavity, oropharynx, meninges, eyeball, and other areas. The number and percentage of cases involved in each location for the two sequences were calculated.

### Quantitative image analysis

Region-of-interest (ROI) analyses of the T1WI-IDEAL images and LAVA-Flex images were conducted by one investigator (JL), who has 15 years of experience in head and neck radiology, at a post-processing workstation. The ROIs were outlined in a circular shape with a size ranging from 4 to 10 mm². Care was taken to avoid cavities, blood vessels, and areas with noticeable artifacts as much as possible when delineating the ROIs.

### Uniformity of fat suppression

The ROI was positioned at four locations in various areas of superficial fat, with one in each quadrant of the image, and repeated on three planes (maxilla, mandible, and clavicle). For each patient and imaging sequence, 12 signal intensity (SI) measurements were acquired. To quantify the uniformity of fat suppression per patient per acquisition, the standard deviation (SD) percentage of these measurements was calculated. The SD percentage was computed using the following formula: dividing the SD of the ROI by the mean of the ROI signal intensities (12).

### Contrast between tumor lesions and subcutaneous fat tissue

The ROI was positioned on the tumor lesion, which included the primary nasopharyngeal tumor and metastatic lymph nodes, as well as the adjacent subcutaneous fat region. This process was repeated three times to calculate the average signal intensity of the tumor lesion and the adjacent superficial fat. The contrast between the tumor lesion (primary nasopharyngeal tumor and metastatic lymph nodes) and fat was determined using the following formula: C_pf_ = (SI_p_-SI_f_)/SI_p_, where C_pf_ represents the contrast between the primary nasopharyngeal tumor and fat, SI_p_ is the mean signal intensity of the primary nasopharyngeal tumor, and SI_f_ is the mean signal intensity of fat. Similarly, for calculating the contrast between metastatic lymph nodes and fat, another formula was used: C_nf_ = (SI_n_ - SI_f_)/SI_n_. Here, C_nf_ stands for the contrast between metastatic lymph nodes and fat, SI_n_ represents the mean signal intensity of metastatic lymph nodes, and SI_f_ denotes the mean signal intensity of fat (12, 21).

### Signal-to-noise ratio (SNR) of tumor and muscle

The SNR of the tumor (SNR_t_) and muscle (SNR_m_) was calculated following the method recommended by the American Society of Electrical Manufacturers (22, 23). The tumor ROI was positioned in non-necrotic areas, while central necrotic areas were avoided if present. For the muscle ROI, it was placed in the lateral pterygoid muscle on the same side as the primary nasopharyngeal tumor. To prevent artifacts, ROIs were placed in the four corners of the image background, away from the patient’s body. The standard deviation (s) and mean value (x) were calculated. The standard deviation (s) and mean value (x) were computed. The noise (N) was determined as N = x/0.66, with 0.66 representing the noise correction factor. Using the measured signal intensity (SI) and N values, the SNR for each sequence’s tumor and muscle was calculated using the equation: SNR = SI/N.

### Statistical analysis

The data analysis was conducted using SPSS for Windows (version 25.0, Chicago, IL). A significance level of P < 0.05 was considered statistically significant. The consistency analysis of interpretations by two radiologists was performed using the Kappa test. A value of k ≤ 0.40 indicates poor consistency, 0.41 ≤ k ≤ 0.75 indicates moderate consistency and k ≥ 0.76 indicates good consistency. For qualitative scale evaluations, the Wilcoxon signed rank test was employed to compare differences between the evaluations of T1WI-IDEAL and LAVA-Flex images. The statistics regarding the number of cases and percentage of tumor involvement in both sequences were presented as frequency and rate, respectively. Comparisons between groups were made using the chi-square test. Regarding quantitative analysis, the paired t-test was utilized to compare differences in all parameters between LAVA-Flex imaging and T1WI-IDEAL imaging.

## Results

### Inter-observer agreement

After performing the Kappa test to assess consistency, the overall image quality demonstrated a Kappa value of 0.808, with a p-value < 0.001, indicating strong agreement. The uniformity of fat suppression yielded a Kappa value of 0.899, with a p-value < 0.001, indicating excellent agreement. The presence of artifacts showed a Kappa value of 0.881, with a p-value < 0.001, indicating high agreement. Additionally, the significance of tumors exhibited a Kappa value of 0.889, with a p-value < 0.001, suggesting substantial agreement. Overall, there was very good consistency in the diagnostic results between the two doctors based on these findings.

### Qualitative image analysis

**Figure 1:**
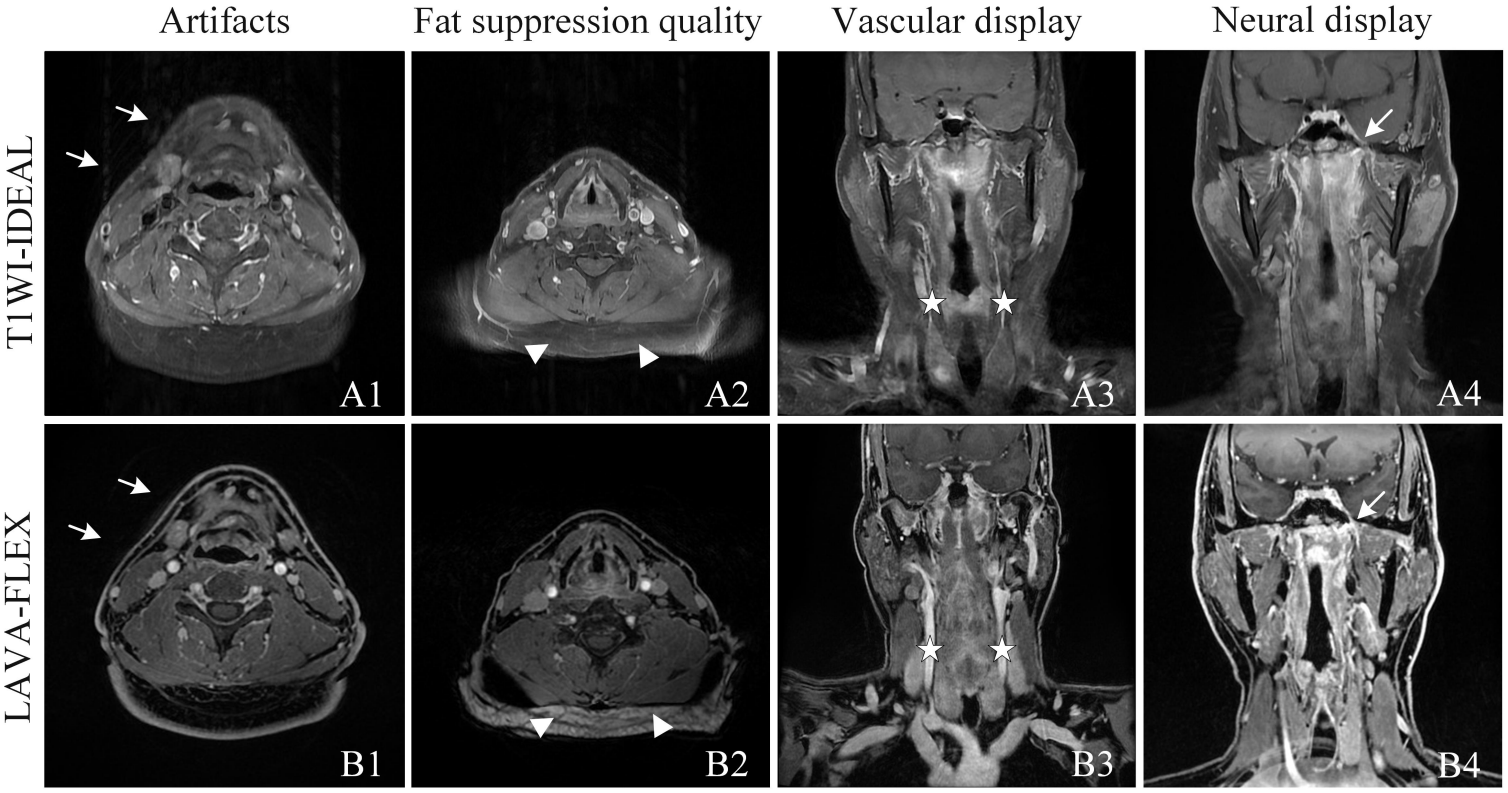
Qualitative analysis of T1WI-IDEAL **(A)** and LAVA-Flex **(B)** images of NPC. Both T1WI-IDEAL and LAVA-Flex MR images demonstrated excellent image quality, receiving a rating of five points. However, both readers noted that the LAVA-Flex sequence exhibited fewer artifacts (**A1**, **B1**, indicated by arrows) and provided superior visualization of vessels (**A3**, **B3**, indicated by pentagrams) and nerves (A4, B4, indicated by arrows) compared to T1WI-IDEAL. On the other hand, T1WI-IDEAL (**A2**) achieved stronger fat suppression (indicated by a triangle) than LAVA-Flex (**B2**).

**Figure 2:**
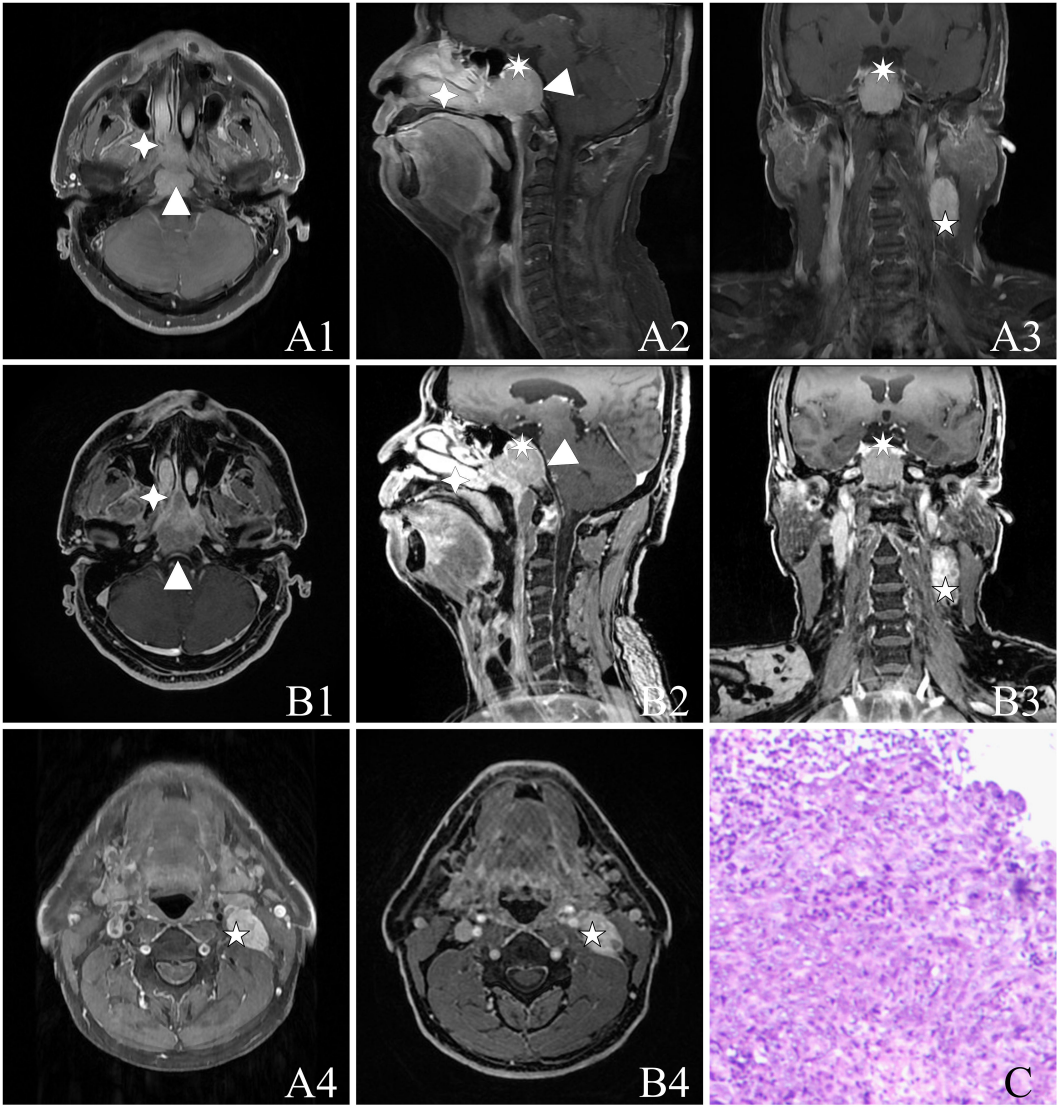
A 55-year-old male with NPC [cT4N1M0, stage IVA, AJCC 8th (24)] with a biopsy pathology of non-keratinizing undifferentiated type of cancer. The figure includes the following image panels: **(A)** T1WI-IDEAL images, consisting of **A1**, **A4** (axial T1WI-IDEAL), **A2** (sagittal T1WI-IDEAL), and **A3** (coronal T1WI-IDEAL). **(B)** LAVA-Flex images, including **B1**, **B4** (axial LAVA-Flex), **B2** (reconstructed sagittal LAVA-Flex), and **B3** (reconstructed coronal LAVA-Flex). **(C)** Hematoxylin and eosin-stained slides depicting the non-keratinizing undifferentiated type of cancer cells at a magnification of HE ×100. Both the T1WI-IDEAL and LAVA-Flex MR images reveal primary foci infiltrating in multiple directions: Forward infiltration is observed in the nasal cavity, nasal septum, right turbinate, bilateral pterygoid process, and pterygopalatine fossa indicated by a four-pointed star; Backward infiltration involves the bilateral posterior pharyngeal space, cephalic longissimus muscle, cervical longissimus muscles, and the pontine anterior pool depicted by a triangle; Upward infiltration is seen in the pterygoid saddles, skull base bony mass, slope, bilateral rupture foramen, cavernous sinus, and pituitary gland represented by a heptagram. Additionally, strings and clusters of metastatic lymph nodes are observed bilaterally in the neck region. Some of these lymph nodes have fused into clusters as indicated by a pentagram.

**Figure 3:**
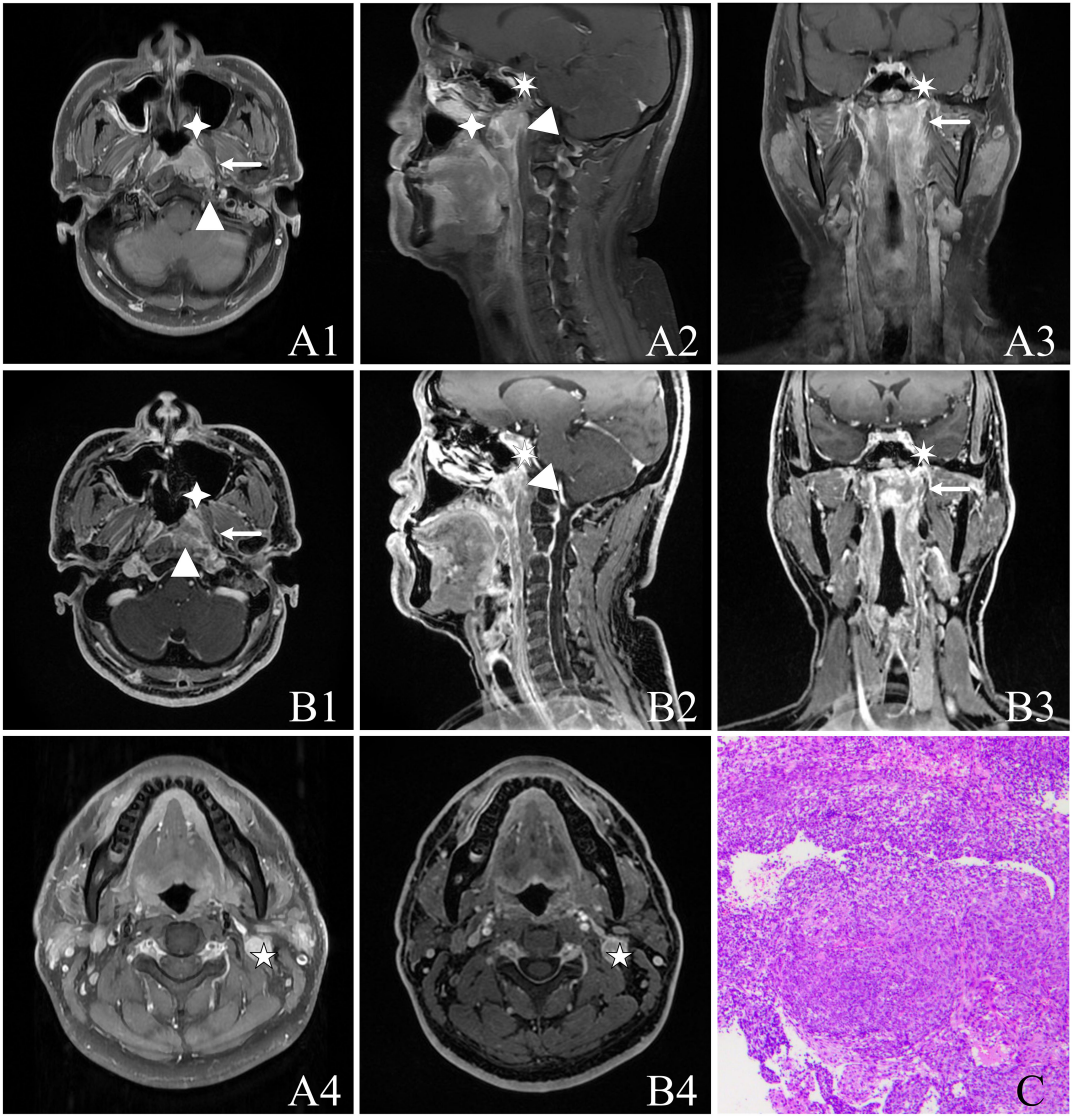
A 37-year-old male with NPC [cT3N1M0, stage III, AJCC 8th (24)] presenting non-keratinizing undifferentiated type of cancer cells on hematoxylin and eosin (H&E) staining (HE ×100). The image panels include **(A)** T1WI-IDEAL images, including **A1**, **A4** (axial T1WI-IDEAL), **A2** (sagittal T1WI-IDEAL), and **A3** (coronal T1WI-IDEAL); **(B)** LAVA-Flex images, including **B1**, **B4** (axial LAVA-Flex), **B2** (reconstructed sagittal LAVA-Flex), and **B3** (reconstructed coronal LAVA-Flex); **(C)** Non-keratinizing undifferentiated type of cancer cells on hematoxylin and eosin (H&E) (HE ×100). Both the T1WI-IDEAL and LAVA-Flex MR images reveal the involvement of NPC in the following areas: Left posterior nasal cavity and nasal septum in the anterior direction (indicated by a four-pointed star); Left cephalic longissimus muscle, cervical longissimus muscle, and anterior intervertebral space in a backward direction (indicated by a triangle); Left parapharyngeal space in an outward direction (indicated by an arrow); Skull base bone in an upward direction (indicated by a heptagram).

Based on the subjective evaluation (see **Table 2**), there was no statistically significant difference in the overall image quality between the two sequences (*P* > 0.05). However, it was observed that the T1WI-IDEAL sequence exhibited better uniformity of fat suppression compared to the LAVA-Flex sequence (*P* < 0.05). Additionally, the LAVA-Flex sequence had fewer artifacts when compared to the T1WI-IDEAL sequence (*P* < 0.05) (see **Figure 1**). Both sequences demonstrated good tumor visualization, and there was no statistically significant difference between them (*P* > 0.05) (see **Figures 2**, **3** and **Table 3**).

**Table 2:** Comparison of qualitative scores between the T1WI-IDEAL and LAVA-Flex sequence images.

Measurement	T1WI-IDEAL [Median (P25, P75)]	LAVA-Flex [Median (P25, P75)]	*P*
Overall image quality	4 (3, 4)	4 (3, 4)	0.371
Fat suppression quality	5 (4, 5)	4 (3, 4)	0.001
Artifacts	3 (3, 3)	4 (3, 4)	0.003
Tumor conspicuity	3 (2, 3)	3 (2, 3)	0.406
Vascular display	2 (2, 3)	4 (3, 4)	0.001
Neural display	2(2, 2)	4(3, 4)	0.001

**Table 3:** Presentation of T1WI-IDEAL and LAVA-Flex sequence on the site of tumor invasion (n, %).

Measurement	T1WI-IDEAL	LAVA-Flex	*P*
Cervical Lymph Nodes	76 (90.5%)	76(90.5%)	NA
Retropharyngeal lymph nodes	51 (60.7%)	51(60.7%)	NA
Cervical metastases	2 (2.4%)	2 (2.4%)	NA
Skull base	45(53.6%)	47(56.0%)	0.757
Cavernous sinus	15 (17.9%)	16 (19.0%)	0.842
Pterygoid sinus	24 (28.6%)	24 (28.6%)	NA
Internal and external pterygoid muscles	23(27.4%)	23(27.4%)	NA
Pterygopalatine fossa	18 (21.4%)	18 (21.4%)	NA
Nasal cavity	30 (35.7%)	30 (35.7%)	NA
Oropharynx	7 (8.3%)	7(8.3%)	NA
Meninges	7(8.3%)	7(8.3%)	NA
Parapharyngeal space	45(53.6%)	47(56.0%)	0.757

### Quantitative image analysis

According to the objective evaluation presented in **Table 4**, it was found that the T1WI-IDEAL sequence demonstrated superior uniformity of fat suppression compared to the LAVA-Flex sequence (*P* < 0.001). On the other hand, the 3D LAVA-Flex sequence exhibited better contrast between the primary lesion and fat (C_pf_), as well as contrast between metastatic lymph nodes and fat (C_nf_), when compared to the T1WI-IDEAL sequence (*P* < 0.001). However, there were no statistically significant differences in the signal-to-noise ratio of tumor (SNR_t_) and signal-to-noise ratio of muscle (SNR_m_) between the two sequences (*P* > 0.05).

**Table 4:** Comparison of the quantitative measurements of the signal intensity between the T1WI-IDEAL and LAVA-Flex sequence images.

Measurement	T1WI-IDEAL	LAVA-Flex	*P*
Fat suppression uniformity assessment			
SI of 12 ROIs	1227.85 ± 407.82	1157.35 ± 921.13	
SD of 12 ROIs	266.92 ± 93.00	510.77 ± 375.16	
SD% f 12 ROIs	22.00 ± 0.06	43.69 ± 0.11	0.000
Contrast assessment			
SI_p_	2924.79 ± 597.97	5650.15 ± 1666.84	
SI_f_	1391.71 ± 403.69	994.15 ± 595.64	
C_pf_	0.52 ± 0.12	0.80 ± 0.16	0.000
SI_n_	2804.01 ± 734.80	5392.7 ± 1410.57	
SI_f_	1391.71 ± 403.69	994.15 ± 595.64	
C_nf_	0.56 ± 0.10	0.81 ± 0.11	0.000
SNR of tumor and muscle			
SNR_t_	86.64 ± 88.86	80.84 ± 139.42	0.707
SNR_m_	53.72 ± 46.21	59.59 ± 101.93	0.594

## Discussion

This study directly compared the LAVA-Flex and T1WI-IDEAL sequences for detecting tumor lesions in NPC. Our findings indicate that the LAVA-Flex sequence provides better image quality with fewer artifacts compared to the T1WI-IDEAL sequence. Moreover, the contrast between tumors (including primary nasopharyngeal foci and lymph node metastases) and fat was significantly improved on the LAVA-Flex sequence. The LAVA-Flex sequence offers a thinner layer thickness of only 1 mm, without layer spacing, making it more suitable for displaying fine structures in the nasopharynx. In contrast, the T1WI-IDEAL sequence requires scanning in axial, coronal, and sagittal directions, which is time-consuming. The LAVA-Flex sequence allows for reconstruction in any orientation within a single scan, thereby saving scanning time. Furthermore, the LAVA-Flex sequence appears to be superior to conventional enhancement sequences in displaying lesions involving the skull base, cavernous sinus, and parapharyngeal space. In conclusion, the LAVA-Flex sequence meets staging requirements for NPC and reduces examination time.

Optimizing staging MRI scanning protocols for patients with NPC has emerged as a prominent research focus in recent times. Discovering more suitable MRI scanning sequences holds paramount importance for clinical endeavors. The intricate anatomy of the head and neck region, coupled with the magnetic field’s inhomogeneity caused by magnetization effects from the paranasal cavity, oral cavity, laryngopharynx, and air/fat interfaces, often leads to geometric distortions, ghosting artifacts, and inadequate fat suppression. Consequently, incorporating MR enhancement scans alongside fat-suppression sequences assumes great significance in NPC staging scans. Skillful implementation of fat suppression not only significantly enhances image quality and lesion detection rates but also furnishes vital information for differential diagnosis. Commonly employed fat suppression techniques encompass the Short T1 Inversion Recovery Sequence (STIR), Spectral Presaturation with Inversion Recovery (SPIR), and Dixon’s technique (Phase Contrast Method, Water-Fat Separation Imaging). Notably, the multipoint Dixon technique offers superior image quality and uniform fat suppression within a shorter scan duration when compared to STIR and spectral presaturation with inversion recovery gadolinium-T1WI techniques (25, 26). Flex technology represents a refined iteration of fat-suppressed imaging, building upon Dixon’s water-lipid separation methodology. 3D LAVA-Flex, an amalgamation of Flex technology and 3D-LAVA, presents a three-dimensional destructive gradient echo pulse sequence recently enhanced with water-fat separation techniques to enhance tissue suppression. This innovative approach has found utility in the screening of abdominal and pelvic lesions, as it facilitates the visualization of both diseased and healthy tissues from diverse perspectives. Moreover, it enables the acquisition of stable images capturing pure water and pure fat within a single expedited scan (11, 27, 28).

In our study, we ventured to utilize the 3D thin-layer high-resolution isotropic LAVA-Flex sequence for the first time in NPC staging scans. Its imaging attributes and advantages manifest in several key aspects. Firstly, the three-dimensional volume acquisition permits scanning with a remarkable thickness of 1mm, thereby yielding images with a high signal-to-noise ratio (SNR). Notably, this isotropic scanning process ensures that pixel dimensions remain uniform across horizontal, vertical, and axial directions—consequently producing cubic-shaped pixels. This feature assumes great significance as it guarantees consistent image resolution regardless of the plane examined (be it axial, sagittal, coronal, or any other orientation), rendering reconstructed images identical to their directly scanned counterparts. By meticulously analyzing both original cross-sectional images and reconstructed counterparts, we can more comprehensively depict the extent of nasopharyngeal tumors, extracapsular invasion patterns, and cervical lymph node metastasis. Furthermore, this technique facilitates better differentiation between tumor tissue and surrounding normal background tissues such as muscles and blood vessels—thus enabling accurate delineation of the interface between tumors and adjacent tissues—an invaluable asset when outlining target tumor areas. Secondly, LAVA-Flex proves particularly adept at displaying cranial nerves—an essential consideration for NPC staging. MRI is widely regarded as the gold standard for visualizing cranial nerves (29). Given that most cranial nerves exhibit slender structures with tortuous paths, conventional T1WI-IDEAL sequences often struggle to fully capture their complexity due to thick and spaced layers. In contrast, LAVA-Flex volumetric imaging scans leverage thin layer thickness, zero spacing, and detailed imaging capabilities to reconstruct cranial nerves with intricate alignments. This approach significantly improves the display rate of cranial nerves through multiplanar reconstruction (MPR) technology, as our research has demonstrated. Moreover, the LAVA-FLEX sequence facilitates the reconstruction of corresponding vascular alignments using maximum density projection (MIP), thereby enhancing our interpretation of the relationship between nerves and adjacent vessels based on superior spatial resolution offered by three-dimensional images. Our study further revealed that the LAVA-Flex sequence outperformed the T1WI-IDEAL sequence in terms of vascularity—an observation consistent with prior investigations focusing on hepatic and carotid arteries (30, 31). Thirdly, LAVA-Flex exhibits fewer artifacts compared to its two-dimensional T1WI-IDEAL counterparts. The T1WI-IDEAL sequence operates on a spin-echo principle, with vascular pulsation artifacts commonly observed in nasopharyngeal scan images. These artifacts arise from the misaligned encoding of spatial information localization caused by vascular pulsations—often manifesting in the direction of phase encoding. In contrast, the LAVA-Flex sequence leverages gradient echo principles and employs flow compensation techniques to mitigate the occurrence of vascular pulsation artifacts. Additionally, our study leveraged auto-calibrating reconstruction for cartesian sampling (ACR) technology parallel imaging technique rooted in k-space domain-eliminating the need for additional coil sensitivity calibration sequences. This streamlines both scanning and processing workflows while reducing artifacts stemming from calibration data. The use of slice-interleaved and phase-encoded techniques significantly enhances scanning speed, diminishes artifacts and bolsters spatial resolution benefits particularly pronounced in three-dimensional accelerated acquisitions. Lastly, the imaging plane benefits from the application of the zero-fill interpolation processing (ZIP) technique-a zero-filling approach that can be employed both between and within slices. Notably, this technique does not increase the actual acquisition matrix but rather improves resolution levels, leading to clearer image details and reduced step-like artifacts during multi-planar reconstruction (MPR) processes.

It is important to recognize in our study that the LAVA-Flex sequence also has certain limitations. Firstly, fat suppression uniformity is less optimal in LAVA-Flex sequences compared to IDEAL sequences. This discrepancy can be attributed to the fact that IDEAL technology acquires an additional echo during scanning, providing better fat suppression capabilities than Flex technology. In regions of the head and neck where anatomical structures exhibit significant variations in shape, such as the supraclavicular region and the floor of the mouth, which are particularly susceptible to uneven magnetic field distribution, there may be magnetic susceptibility artifacts present within the LAVA-Flex images, leading to inadequate fat suppression. In contrast, T1WI-IDEAL sequences can effectively avoid fat suppression failures in these specific layers. Secondly, the LAVA-Flex sequence may exhibit folding artifacts in the head-to-tail direction. This limitation arises from the nature of the 3D sequence itself, as phase encoding in the scanning plane direction can result in image folding at both ends. To mitigate this issue, increasing the number of slices and expanding the scanning range can be employed to ensure that there are no folding artifacts within the diagnostic range (from the middle of the temporal lobe to the aortic arch). Thirdly, the measurement differences in tumor and metastatic lymph node sizes were not directly compared between the two sequences in our study. Further investigations are warranted in this particular aspect. Lastly, it is worth mentioning that in our study design, we acquired the T1WI-IDEAL sequence followed by the LAVA-Flex sequence after administering a contrast agent. Therefore, changes in gadolinium concentration post-injection may impact enhancement levels. Nevertheless, we anticipate any resulting differences to be minimal. In our future research, we plan to incorporate cutting-edge deep learning techniques such as MDSN, OIF-Net, UG-Net, and DeepMTS to enhance the image quality even further. We will also investigate their potential in diagnosing NPC and predicting patient survival (32–36).

In conclusion, both the T1WI-IDEAL sequence and the LAVA-Flex sequence yielded satisfactory images in MRI-enhanced scans for staging NPC. Although the fat suppression uniformity of the LAVA-Flex sequence is not as optimal as that of the T1WI-IDEAL sequence, it offers notable advantages such as reduced scanning time, effective control of motion and flow artifacts, and excellent visualization of nerves and vessels. Moreover, with thin-slice isotropic scanning, image reconstruction can be performed in any desired plane, allowing for a comprehensive display of the primary nasopharyngeal tumor and detailed information regarding cervical lymph node metastasis. The clinical significance of the LAVA-Flex sequence in accurately staging NPC and guiding subsequent treatment plans cannot be overstated. Therefore, its wider implementation in MRI scanning for NPC staging is highly recommended.

## Data availability statement

The raw data supporting the conclusions of this article will be made available by the authors upon request for legitimate purposes, such as research or academic uses. Interested parties may contact the corresponding author to obtain the necessary information. The authors are committed to providing the data without undue reservation, ensuring that it is accessible to those who need it.

## Ethics statement

The studies involving humans were approved by the Medical Ethics Committee of Clinical Oncology School of Fujian Medical University, Fujian Cancer Hospital. The studies were conducted in accordance with the local legislation and institutional requirements. The participants provided their written informed consent to participate in this study.

## Author contributions

LP: Conceptualization, Funding acquisition, Project administration, Resources, Validation, Visualization, Writing – original draft. BC: Formal analysis, Funding acquisition, Project administration, Resources, Writing – original draft. EY: Data curation, Visualization, Writing – review & editing. YL: Data curation, Investigation, Writing – original draft. JL: Data curation, Investigation, Software, Supervision, Writing – original draft. DZ: Data curation, Investigation, Writing – original draft. YF: Data curation, Investigation, Writing – original draft. ZC: Data curation, Investigation, Writing – original draft. HZ: Data curation, Investigation, Writing – original draft. YC: Conceptualization, Writing – review & editing. ZZ: Conceptualization, Project administration, Writing – original draft, Writing – review & editing.
